# Feasibility of Robotic-Assisted Laparoscopic Nephroureterectomy in Left Ventricular Assist Device Patient

**DOI:** 10.1155/2012/282680

**Published:** 2012-10-10

**Authors:** Tariq A. Khemees, Ahmad Shabsigh

**Affiliations:** Department of Urology, The Ohio State University Wexner Medical Center, Columbus, OH 43212, USA

## Abstract

Left ventricular assist devices (LVADs) have revolutionized management options for patients with advanced heart failure. It is not uncommon for patients treated with these devices to present with noncardiac surgical conditions including urologic problems. Maintaining perioperative hemodynamic and hematologic stability is a special challenge. The minimally invasive surgery provides well-documented advantages over the open approach including a less operative blood loss and faster convalescence. In carefully selected patients, robotic-assisted surgery can be utilized in the management of patients with complex urologic diseases in a dire need for these benefits. We present the first case of robotic-assisted laparoscopic nephroureterectomy (RANU) with retroperitoneal lymph node dissection for upper tract transitional cell carcinoma (TCC) in a patient treated with LVAD.

## 1. Introduction 

Nephroureterectomy with bladder cuff excision is the standard of care for high-grade and invasive TCC [1]. The procedure can be performed via an open or minimally invasive laparoscopic approach with similar oncologic outcomes [2]. However, management of the distal ureter and bladder cuff remains the biggest challenge of laparoscopic technique [3, 4]. Several authors have reported an easier excision of the distal ureter and bladder cuff, in addition to a less blood loss and faster recovery when the robotic approach was used [5–8].

Since approval by the Food and Drug Administration (FDA) as a bridge to heart transplant in 2008 and as destination therapy in 2010, the HeartMate II LVAD continues to provide an effective mode of treatment for the growing population of advanced heart failure patients [9]. With this in mind, increasing number of LVAD-treated patients has been presented with surgical conditions [10, 11]. Due to the risk of device thrombosis, these patients need to stay on anticoagulation during and after surgery [12], which carriers the risk of increased operative blood loss. Pneumoperitoneum and location of the LVAD may lead to unique intraoperative challenges. We present the first case of RANU for upper tract TCC in LVAD-treated patient focusing on intraoperative management and short-term outcomes.

## 2. The Case

### 2.1. Patient Presentation

**Figure 1:**
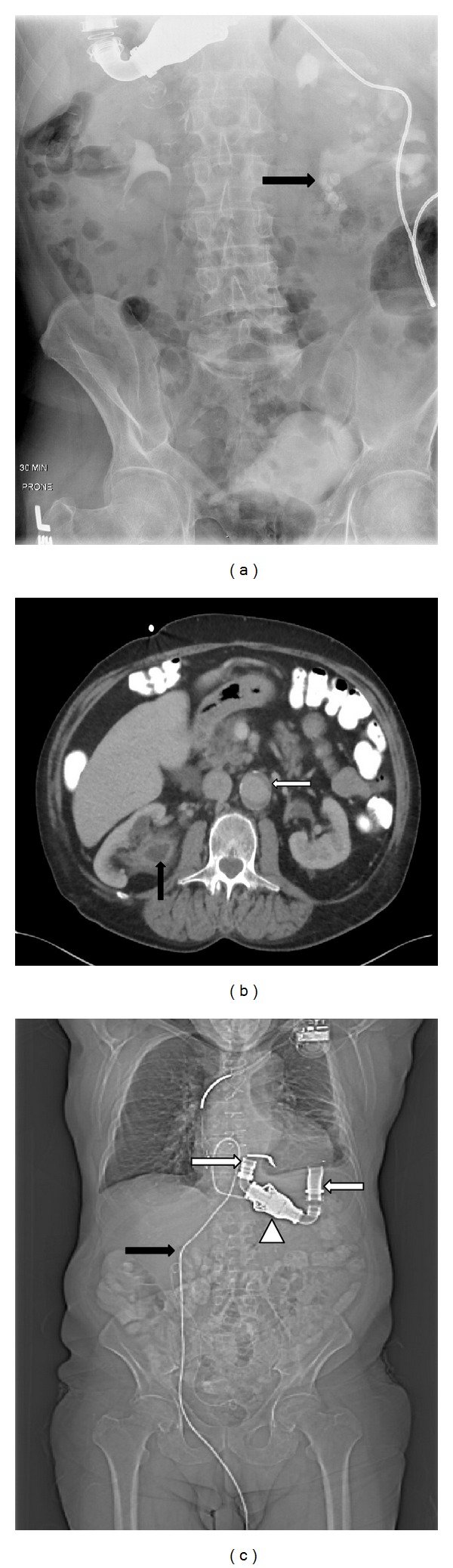
(a) Intravenous pyelogram image showing multiple right renal pelvis stones (black arrow). (b) Axial CT image at the level of the renal hilum showing incidental aortic aneurysm (white arrow) and right renal pelvis filling defect representing urothelial carcinoma (black arrow), and (c) scout CT image showing the location of LVAD cannulae (white arrows), continuous flow pump (white arrow head), and percutaneous connection cord (black arrow).

A 71-year-old Caucasian male presented with the New York Heart Association (NYHA) class III heart failure managed with HeartMate II LVAD as destination therapy and implantable cardioverter defibrillator (ICD). Following right renal colic, the patient underwent intravenous pyelogram at an outside institution which demonstrated right renal pelvic stones (Figure 1(a)). During ureteroscopic laser lithotripsy, multiple lesions were discovered in his right renal pelvis. Biopsy revealed high-grade urothelial carcinoma. The patient was then referred to our institution 9 months following his cancer diagnosis for further management. CT abdomen was performed and showed multiple filling defects in the right renal pelvis, enlarged retroperitoneal lymph nodes, incidental 4.9 cm abdominal aortic aneurysm, and no evidence of tumor metastases (Figure 1(b)). The aneurysm was asymptomatic and did not meet size criteria for a prophylactic repair. 

Medical history includes atrial fibrillation, hypertension, hyperlipidemia, and diverticular disease. Surgical history includes bilateral open nephrolithotomy, repair of bilateral inguinal hernia, and cataract surgery.

The patient was counseled regarding management options for TCC. Being a high surgical risk, he was offered minimally invasive endoscopic laser resection; however he elected to undergo nephroureterectomy with bladder cuff excision. A conference was held with a team of cardiac anesthesiologist, cardiologist, perfusionist, and the managing urologist. Hemodynamic effects of pneumoperitoneum and risks of surgery were discussed. We detailed to the patient the risks of the periprocedural complications. After informed consent, RANU with bladder cuff excision and retroperitoneal lymph node dissection was planned. We thought the robotic approach may help minimize blood loss and expedite recovery.

### 2.2. Procedure and Technique

Two days prior to surgery, the patient was bridged from warfarin to 5000 IU subcutaneous heparin 6 hourly while continuing his aspirin therapy. International normalized ratio (INR) was 2 at time of surgery. Intraoperative monitoring plan included establishing an arterial line, central venous line, pulmonary artery catheter, transesophageal echocardiography (TEE), esophageal temperature probe, electrocardiography, pulse oximetry, and serial arterial blood gas (ABG) measurements. Cardiac defibrillator pads were placed and the ICD was turned off preoperatively. Standard anesthetic agents were used for induction. A single dose of 1.5 gm intravenous vancomycin and two doses of 4.5 gm *Zosyn* (piperacillin and tazobactam) were given. Flexible cystoscopy was performed to rule out bladder neoplasm. The patient was repositioned into left lateral position and the operating table was gently flexed to increase space for port placement. The location of the LVAD and its percutaneous connection cord was determined by physical examination and preoperative CT images (Figure 1(c)).

**Figure 2:**
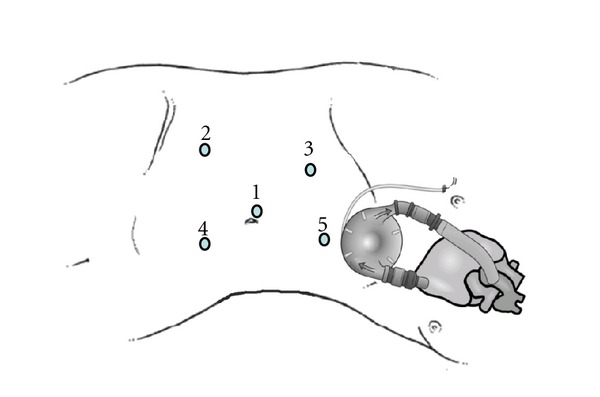
Schematic drawing depicting placement of ports during robotic-assisted laparoscopic nephroureterectomy in relation to LVAD position. Port 1 : 12 mm robotic telescope port, port #2 : 8 mm robotic instrument port, Port 3 : 8 mm robotic instrument port, Port 4 : 12 mm surgical assistant port, Port 5 : 5 mm surgical assistant port used for liver retraction. Ports 2 and 3 were used for the right and left robot arms, respectively, during the nephrectomy part of the surgery. For the distal ureter and bladder cuff excision, the left robotic arm was repositioned to port 2 and the right robotic arm was repositioned to port 4.

A 15 mmHg pneumoperitoneum was obtained via Veress needle CO_2_ gas insufflation. The 12 mm port was placed under direct vision using standard laparoscope. We placed an 8 mm robotic instrument port in the right lower quadrant avoiding inferior epigastric vessels. A second 8 mm port was placed in the right upper quadrant. A 12 mm assistant port was placed inferior and to the left of the umbilicus and another 5 mm assistant port was placed in the epigastric area for liver retraction. All ports were placed under direct vision and attention was paid not to damage the LVAD or its connections (Figure 2). The robot, da Vinci S system (Intuitive Surgical, Inc. Sunnyvale, CA, USA), was docked and standard nephroureterectomy was performed. Briefly, the ascending colon was mobilized medially and the duodenum was mobilized off the vena cava to visualize the renal hilum. The renal artery was controlled using Hem-o-Lok ligation system while the renal vein was controlled with Endo GIA instruments. A Hem-o-Lok clip was placed around the distal ureter to prevent tumor spillage and dissection was carried down to the pelvic brim. At this point the robot arms were repositioned [6] and access to the pelvis was obtained by shifting the left robotic arm from port 3 to port 2, and the right robotic arm from port 2 to port 4 (Figure 2). The bladder cuff was dissected along with the ureter and the defect was closed in a double-layer running fashion using 3-0 Polysorb sutures. The surgical specimen with the pericaval and hilar lymph nodes were placed in Endo Catch retrieval bag and extracted through an extended 12 mm mid-line port incision. All ports were closed in a multilayer fashion and a pelvic drain was inserted on the right side. Total operative time was 230 minutes and estimated blood loss was 200 mL.

Postoperative course was uncomplicated and the patient was discharged on postoperative day 7 after reinstating his warfarin therapy. Cystogram done on postoperative day 13 demonstrated no extravasation. Pathology report revealed T2 N1 high-grade TCC with negative surgical margins. Unfortunately, a year after surgery, the patient developed disease progression but is still alive. Written informed consent was obtained from the patient for publication of this case report/any accompanying images. A copy of the written consent is available for review by the Editor-in-Chief of this journal.

## 3. Discussion

A multidisciplinary team approach was used to manage TCC in LVAD-treated patient. Several mechanisms of bleeding have been described in patients treated with these devices [12, 13]. The intraoperative risk of bleeding was assessed against the need for continued anticoagulation to prevent pump thrombosis; as such, we elected to proceed with the robotic approach in an attempt to lower operative blood loss and minimize its effect on cardiac output while cardiology team planned anticoagulation and a perfusionist was available throughout surgery to monitor for effective cardiac output.

Anesthetic challenges stem from the need of knowledge and monitoring for physiologic derangement during induction of anesthesia, positioning, and surgery. Adjusting LVAD parameters to meet any acute hemodynamic changes and maintaining safe cardiac output throughout surgery is a unique situation. The HeartMate II LVAD console provide real-time estimate of four parameters: speed in revolutions per minute, pump flow (PF) in liter per minute, pulsatility index (PI), and power consumption in Watts. The interpretation of each parameter has been previously detailed [9]. The baseline PF and PI in our patient prior to induction of general anesthesia were 4.5 L/min and 5, respectively. At time of pneumoperitoneum, the PI dropped from 5 to 3 and was promptly managed with crystalloid and colloid fluid administration. The TEE finding played a critical role in assessing preload, monitoring for acute change in ventricular volumes, and guiding fluid administration. PF was used as a surrogate for estimated cardiac output. Inotropic support was not required throughout surgery.

Several other factors must be considered when planning surgery on LVAD-treated patients. First, perioperative antibiotic regimen is needed to lower the risk of device infection. Previous reports have shown that in the advent of LVAD colonization with an infectious agent, it would not be possible to eradicate the pathogen and device replacement might be inevitable [14]. Additionally, if a cardiovascular collapse occurred in LVAD patient, current guidelines recommend following the advanced cardiac life support protocol in term of medications and shock management; however, chest compressions should not be performed as might lead to dislodgement of LVAD cannulae [15]. We placed defibrillator chest pads after switching off the ICD preoperatively.

By presenting this case and its management, we hope that LVAD patients would not be denied access to definitive therapeutic surgical option for urologic conditions based solely on their cardiac risk. In this case, a delay in patient's presentation for surgical therapy may have contributed to his cancer progression. Of note, we do not advocate that all cases to be managed in similar fashion but rather approached individually. The surgery was performed by experienced robotic surgeon and was done at the operating room located in a specialized heart hospital at our institution. Intensive monitoring and experienced cardiac and anesthesia teams were available throughout the surgery. A multidisciplinary team approach is the key to the patient safety and successful outcomes.

## 4. Conclusion

With identification and maintenance of hemodynamically safe operative zone, RANU is a feasible option for management of high-grade TCC in patients managed with LVAD. The low intraoperative blood loss minimized hemodynamic derangement during surgery.

## Abbreviations

CT: Computed tomography
ICD: Implantable cardioverter defibrillator
LVAD: Left ventricular assist devices
PF: Pump flow
PI: Pulsatility index
RANU: Robotic-assisted laparoscopic nephroureterectomy
TCC: Transitional cell carcinoma
TEE: Transesophageal echocardiography.
